# Identification of PLK1 as a New Therapeutic Target in Mucinous Ovarian Carcinoma

**DOI:** 10.3390/cancers12030672

**Published:** 2020-03-13

**Authors:** Roberta Affatato, Laura Carrassa, Rosaria Chilà, Monica Lupi, Valentina Restelli, Giovanna Damia

**Affiliations:** 1 Laboratory of Molecular Pharmacology, Istituto di Ricerche Farmacologiche Mario Negri-IRCCS, Via Mario Negri 2, 20156 Milan, Italy; roberta.affatato@marionegri.it (R.A.); laura.carrassa@unifi.it (L.C.); rosaria.chila@ifom.eu (R.C.); monica.lupi@marionegri.it (M.L.); r.vale90@hotmail.it (V.R.); 2 Core Research Laboratory—ISPRO, Viale Pieraccini 6, 50139 Firenze, Italy; 3 Istituto FIRC di Oncologia Molecolare (IFOM)—Via Adamello 16, 20139 Milan, Italy

**Keywords:** screening, PLK1, mucinous ovarian carcinoma

## Abstract

Mucinous epithelial ovarian cancer (mEOC) is a rare subset of epithelial ovarian cancer. When diagnosed at a late stage, its prognosis is very poor, as it is quite chemo-resistant. To find new therapeutic options for mEOC, we performed high-throughput screening using a siRNA library directed against human protein kinases in a mEOC cell line, and polo-like kinase1 (PLK1) was identified as the kinase whose downregulation interfered with cell proliferation. Both PLK1 siRNA and two specific PLK1 inhibitors (onvansertib and volasertib) were able to inhibit cell growth, induce apoptosis and block cells in the G2/M phase of the cell cycle. We evaluated, in vitro, the combinations of PLK1 inhibitors and different chemotherapeutic drugs currently used in the treatment of mEOC, and we observed a synergistic effect of PLK1 inhibitors and antimitotic drugs. When translated into an in vivo xenograft model, the combination of onvansertib and paclitaxel resulted in stronger tumor regressions and in a longer mice survival than the single treatments. These effects were associated with a higher induction of mitotic block and induction of apoptosis, similarly to what was observed in vitro. These data suggest that the combination onvansertib/paclitaxel could represent a new active therapeutic option in mEOC.

## 1. Introduction

Epithelial ovarian cancer (EOC) is the most lethal gynecological cancer, with about 239,000 new cases and 152,000 associated deaths worldwide annually [1,2]. Despite significant advances in surgical procedures and chemotherapy over the last two decades, the five year survival rate is lower than 50% for all stages and approximately 25% for advanced stage patients [3]. The high mortality is due to late diagnosis and primary or acquired resistance to therapy [4]. While histological and molecular data have clearly demonstrated that EOC is a heterogeneous disease, patients are still treated as a homogeneous group with cyto-reductive surgery followed by platinum/taxane doublet therapy [5,6]. Amongst the five histotypes (high-grade serous (ovarian cancer)—HGSOC, clear-cell, endometrioid, mucinous and low-grade serous), mucinous epithelial ovarian cancer (mEOC) represents approximately 3% of EOC and is classified as a Type I (low grade) tumor [7,8]. Most patients (80%) with mEOC are diagnosed at an early stage and have a good prognosis; however, advanced mEOC (Stage III/IV) has a very dismal one, with a median overall survival (OS) of 12–14 months, well below the 37–42 months OS observed in patients with non-mucinous epithelial ovarian cancer [5,7]. This is probably due to the fact that mEOC is poorly responsive to platinum-based chemotherapy compared to the other EOC histological subtypes [5,9]. In fact, only ≈30% of patients respond to first line therapy, whereas 70% of high-grade serous ovarian cancer (HGSOC) patients do [10,11]. mEOC is also different from other subtypes at the molecular level. In fact, only 16–52% of mEOCs have a *TP53* mutation compared to 99% of HGSOCs [12,13]. mEOC is not associated with *BRCA1/2* mutations, whereas approximately 25% of HGSOCs carry either germline or somatic mutations of these genes [12,14]. *KRAS* mutations are observed in 40–50% of patients, and *ERBB2* (human epidermal growth factor receptor 2, HER2) gene amplification has been observed in 20–30% of invasive mEOCs [15,16]. Furthermore, mEOC has been associated with a homozygous loss of the cyclin-dependent kinase inhibitor 2A (*CDKN2A*) locus, and mutations in genes that activate the mitogen activated protein kinase (MAPK) pathways (such as *BRAF*, *AKT1* and *PI3K*) [13]. 

A better understanding of mEOC biology and more specific and active treatments are required to improve the prognoses of these patients. However, the rarity of the disease renders the preclinical and clinical research on this tumor type difficult [12]. Recent improvements in high-throughput screening and the availability of RNA interference (RNAi) libraries have demonstrated the power of this approach for target discovery and validation [17,18]. With the aim of identifying new therapeutic targets in mEOC, we performed high-throughput screening with a piece of technology validated in our laboratory [19] and a siRNA library targeting 719 human kinases in a mEOC cell line. We were able to identify polo-like kinase 1 (PLK1), a mitotic serine/threonine kinase, as a potential therapeutic target in mEOC. We explored the effects of PLK1 inhibitors in mucinous ovarian cancer cell lines, either as single agents or in combination with the chemotherapeutic drugs used as standard therapy for treatment of mEOC, and found that the combination of onvansertib (a PLK1 inhibitor) and paclitaxel was extremely active both in vitro and in vivo.

## 2. Materials and Methods 

### 2.1. Cell Culture and Drugs 

MCAS cells were grown in MEM (minimum essential medium) with 1% glutamine and 20% fetal bovine serum. EFO27 was grown in RPMI1640 with 1% glutamine and 10% fetal bovine serum. JHOM1 was grown in DMEM/HamF12 with 1% glutamine, 10% fetal bovine serum and 0.1 mM NEAA. All cell lines were maintained in a humidified 37 °C incubator with 5% CO_2_. Cell lines were obtained from ATCC, and their authentication has been carried out by the authors within the last 6 months.

The PLK1 inhibitors volasertib (Selleckchem) and onvansertib (kindly provided by Trovagene, Inc, San Diego, LA USA) were dissolved in DMSO as 10 mM stock solutions and stored at −20 °C for in vitro studies. For in vivo studies, onvansertib was dissolved in vehicle (0.5% methylcellulose Tween-20), freshly, on days of dosing. Cisplatin (CDDP, Sigma-Aldrich, Milan, Italy) was dissolved in medium before use. Paclitaxel (Indena S.p.a., Milan, Italy) was dissolved in DMSO for in vitro studies and stored at −20°; for in vivo studies it was dissolved in 50% CremophorEL (Sigma-Aldrich, St. Louis, MO, USA) and 50% ethanol, and further diluted with saline before use [20]. A list of all the drugs used and their mechanisms of action is provided in Table S1.

### 2.2. High-Throughput siRNA Screening 

The Mission siRNA Human Kinase Panel (Sigma Aldrich, St Louis, MO, USA) comprising 719 different targets, represented in three different siRNAs, was used. The lyophilized siRNAs were dissolved to a final concentration of 2 µM in nuclease free water and the siRNAs targeting the same gene were pooled at equal molarity in 96-well plates. For the high-throughput screening, we used an automated liquid handling system (JANUS^TM^, PerkinElmer, Waltham, MA, USA), connected to a WinPREP for Janus software. MCAS cells were seeded at 25,000 cell/mL (25 µL/well) in 384 well plates; the next day, cells were transfected with the siRNA pool (60 nM) of each target or mission siRNA universal negative control included in the library. Lipofectamine 2000 (0,05 µL/well) was used as transfection reagent. Ninety-six hours after transfection, cell survival was analyzed by MTS assay (Promega, Madison, Wisconsin, USA). MTS reagent (5 µL) was added to the cells, and after a constant incubation time for all the plates, absorbance was acquired using a plate reader (Infinite M200, TECAN, Mannedorf, Switzerland). Each sample was transfected in triplicate, and the entire screening was repeated twice. The validation procedure was performed as follows: each of the three siRNAs of the pool was deconvoluted and transfected singly. A second validation was performed with specific esiRNAs (endoribonuclease-prepared siRNAs, Sigma Aldrich, St Louis, MO, USA) transfected with Lipofectamine 2000 (Invitrogen, Carlsbad, CA, USA) at a concentration of 50 nM, as already reported [20]. esiRNAs are a mixture of siRNA oligos targeting the same mRNA sequence, leading to highly specific and effective gene silencing. Negative control esiRNA was EGFP esiRNA. 

### 2.3. Western Blot Analysis 

Cells were lysed in ice-cold whole cell extract buffer containing 50 mM TrisHCl at pH 7.4, 250 mM NaCl, 0.1% Nonidet NP40, 5 mM EDTA and NaF 50 mM with a protease inhibitor cocktail (Sigma). Lysates were cleared by centrifuging at 12,000 rpm for 5 min. Cell lysates containing equal amounts of protein (30–70 µg) were resolved on 10–12% SDS-PAGE (polyacrylamide gel electrophoresis) gels. The proteins were then transferred to nitrocellulose membranes (PROTRAN, Schleicher and Shull). Immunoblotting was carried out with the following antibodies and visualized using Odissey FC Imaging System (Li-COR): anti-PLK1 (F-8) #sc17783, anti-actin (C-11) #sc1615 and anti-beta tubulin #sc9104 were provided by Santa Cruz Biotechnology. Anti-phospho-Histone H3 (Ser10) (6G3) #9706 was purchased from Cell Signaling Technology and anti-H2AX pSer139 #05-636 was purchased by Millipore. The horseradish peroxidase (HRP) conjugated secondary antibody anti-goat (sc-2354) was purchased from Santa Cruz Biotechnology (Heidelberg, Germany). The anti-rabbit (#1706515) and anti-mouse (#1706516) antibodies were purchased from BIO-RAD LABORATORIES S.r.l.

### 2.4. Cell Viability Assay 

To validate the effect of PLK1 downregulation in mucinous ovarian cancer cell lines, MCAS and EFO27 were seeded in 96 well plates, and after 24 h were transfected with specific esiRNA of PLK1 (50nM) by Lipofectamine 2000. Cell viability was measured 48 and 72 h after transfection by MTS assay. To evaluate the cytotoxic effect of PLK1 inhibitors, cell lines were seeded in 96 well plates at densities of 10,000 cell/mL for MCAS and JHOM1, and 15,000 cell/mL for EFO27 in 10% fetal bovine serum medium. After 48 h, cells were treated with serial dilution of onvansertib or volasertib, and 72 h later cell viability was assessed by MTS assay. Drug–dose response curves were generated and IC50 calculated by using Prism7.05 (GraphPad Software, San Diego, CA, USA). To assess the effect of the combination of PLK1 inhibitors (onvansertib and volasertib) with cisplatin and/or paclitaxel and/or eribulin and/or PI3K inhibitor (PIK75), cells were seeded, and 48 h after, treated simultaneously with a growing concentration of the drugs, and cell viability was measured 72 h after by MTS assay. Results were examined by isobologram analysis with Calcusyn Software (Biosoft, Cambridge, UK), and combination index (CI) values at the IC_50_ were calculated to assess the efficacy of the combination, as already reported [21]. All the experiments were performed in triplicate and repeated at least twice.

### 2.5. Flow Cytometry 

For flow cytometric analysis of DNA content, cells were fixed in ice-cold 70% ethanol; washed in PBS; resuspended in 1 mL of 25 μg/mL of propidium iodide and 12.5 μL of RNase (1 mg/mL); and stained for 2h at room temperature in the dark. Cell cycle analysis was done on at least 10,000 cells for each sample using the FACS Calibur (Becton Dickinson, Franklin Lakes, NJ, USA) [21]. 

For two-parameter flow cytometry analysis of DNA content and p-S10 histone H3, about 1 × 10^6^ cells fixed in ethanol 70% were washed with PBS and permeabilized in Triton X-100 0.25% in PBS for 10 min on ice. Then, cells were washed and incubated with 100 μL of anti p-S10 histone H3 (Cell Signaling Technology #9706) diluted 1:100 in PBS containing 0.5% BSA for 2 h at room temperature.

After washing with PBS, cells were incubated with Alexa-fluor488 (goat anti-mouse, Molecular Probe #A-11017) diluted 1:500 in PBS + 0.5% BSA for 1 h at room temperature. After the incubation with antibody, cells were centrifuged, resuspended in 2.5 μg/mL PI in PBS plus 25 μL of 1 mg/mL RNase in water, incubated overnight and analyzed.

### 2.6. Caspase-3 Activity Assay 

Caspase-3 activity was measured by an enzymatic assay using a fluorogenic substrate for caspase-3, Ac-DEVD-AMC (acetyl Asp-Glu-Val-Asp 7-amido-4-methylcoumarin). MCAS protein extracts were collected after 24 and 48 h of treatment with onvansertib 15 nM, paclitaxel 2 nM or both. They were mixed with the apoptosis buffer (Hepes pH 7.5 20 mM, glycerol 10%, DTT 10 mM) in a white 96-well plate and incubated at 37 °C for 5 min. The substrate was then added at a final concentration of 12.5 µM. Fluorescent AMC production was measured at excitation 370 nm and emission 460 nm wavelengths, using a plate reader (Infinite M200, TECAN). The caspase-3 activity of each sample was examined in duplicate and expressed as the linear change in fluorescence units per hour and normalized for the protein concentration. 

### 2.7. Xenograft Model 

Five-week-old female NCr-nu/nu mice were obtained from Envigo Laboratories (Italy), and maintained under specific pathogen-free conditions, housed in isolated vented cages and handled using aseptic procedures. Procedures involving animals were conducted in conformity with the following laws, regulations and policies governing the care and use of laboratory animals: Italian Governing Law ( Decreto Legge 26/2014; authorization number 19/2008-A issued 6 March 2008 by the Ministry of Health); Mario Negri Institutional Regulations and Policies providing internal authorization for persons conducting animal experiments (Quality Management System Certificate: UNI EN ISO 9001:2008, regulation number 6121); the NIH Guide for the Care and Use of Laboratory Animals (2011 edition); and the EU directive and guidelines (EEC Council Directive 2010/63/UE). An institutional review board and the Italian Ministry of Health approved all the in vivo experiments performed with PDXs. Exponentially growing MCAS cells (approximately 5 × 10^6^ cells per mouse) were injected subcutaneously. Animals were randomized nine/group when tumors reached approximately 120 mg in different experimental groups (control, onvansertib, paclitaxel and combination). Onvansertib was given orally at the dose of 50 mg/kg for 4 consecutive days for 3 cycles with a 3 days rest, while paclitaxel was administered intravenously at the dose of 20 mg/kg once a week for 3 weeks. In the combined treatment, paclitaxel was administered 2 h after the last onvansertib treatment dose. Tumor growth was measured twice weekly with a Vernier caliper, and tumor weights (mg = mm^3^) were calculated as follows: (length (mm) × width^2^ (mm^2^))/2 where width<length); body weights were registered and considered an indirect parameter of drug toxicity. The efficacy of treatment was evaluated using the best tumor growth inhibition (%T/C = (mean tumor weight of treated tumors/mean tumor weight of control tumors) × 100). A T/C value < 42% is considered the minimum for antitumoral activity, according to published criteria [22]. For pharmacodynamic studies, tumor bearing mice were treated with single or combined drugs following the schedule, doses and routes used in the experiment; however, mice were treated for just one cycle and then sacrificed 24 h after the last treatment with onvansertib in one group, and 24 h after paclitaxel treatment in both paclitaxel single treatment group and in the combination groups. The tumors were removed and snap-frozen. The frozen samples were homogenized in protein lysis buffer, loaded on SDS-PAGE, and immunoblotted. 

### 2.8. Statistical Analysis 

Statistical significance was determined with GraphPad Prism7.05 (GraphPad Software, San Diego, CA, USA). Figures legends specify which test was used. 

### 2.9. Ethics Approval and Consent to Participate

Procedures involving animals were conducted in conformity with the following laws, regulations and policies governing the care and use of laboratory animals: Italian Governing Law (Decreto legge 26/2014; authorization no.19/2008-A issued 6 March 2008 by the Ministry of Health); Mario Negri Institutional Regulations and Policies providing internal authorization for persons conducting animal experiments (Quality Management System Certificate: UNI EN ISO 9001:2008, regulation number 6121); an institutional review board and the Italian Ministry of Health approved all the in vivo experiments performed with PDXs (project authorization #9F5F5.69.EXT28). 

## 3. Results

### 3.1. High-Throughput siRNA Screening Identified PLK1 as a Potential Target 

**Figure 1:**
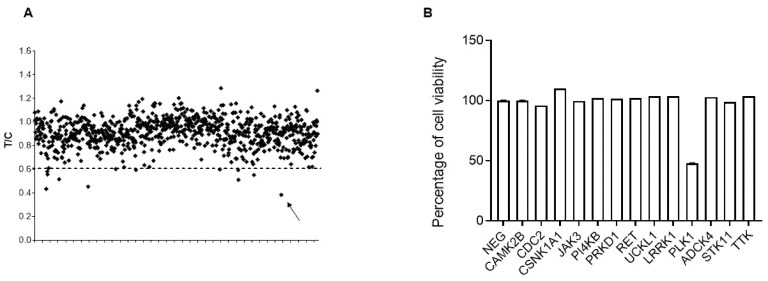
High-throughput siRNA screening in the mucinous epithelial ovarian cancer (mEOC) cell line MCAS. (**A**) Summary of the effects of downregulation on each member of the 719-in-size target pool of the siRNA library. Data are plotted as ratios of transfected siRNA/negative control siRNA mean absorbance values (T/C) and represent the means of the independent siRNA screenings done. The dashed line selected 17 siRNA with a T/C ≤ 0.6. The arrow underlines the T/C value of PLK1. (**B**) Validation of siRNA screening. MCAS cell survival after 96 h of transfection using specific esiRNA against positive hits identified in the screening.

We performed a high-throughput screening using a siRNA library targeting 719 human kinases in the MCAS cell line, and the effect of downregulation of each individual siRNA on cell growth was calculated by dividing the mean absorbance in the three replica wells transfected with siRNA (treated sample-T) by the mean absorbance of the replica wells transfected with a control/negative siRNA (control sample-C) (T/C ratio). Two independent experiments were run and a preselected, arbitrary T/C cut-off value of 0.6, corresponding to 40% of inhibition of cell growth was chosen. Following those criteria, 17 hits with a mean of T/C ≤ 0.6 in the two experiments were found (Figure 1A, Table S2). We then validated 13 out of the 17 positive hits with specific esiRNAs, and only polo-like kinase1 (PLK1) esiRNA, a mitotic serine/threonine kinase, was able to significantly interfere with cell survival (Figure 1B). Concerning the two other members of the polo-like kinase family, PLK2 and PLK3, they had no effect on cell survival (T/C > 0.6). 

### 3.2. The Effect of PLK1 Inhibition in Mucinous Ovarian Cancer Cell Lines 

**Figure 2:**
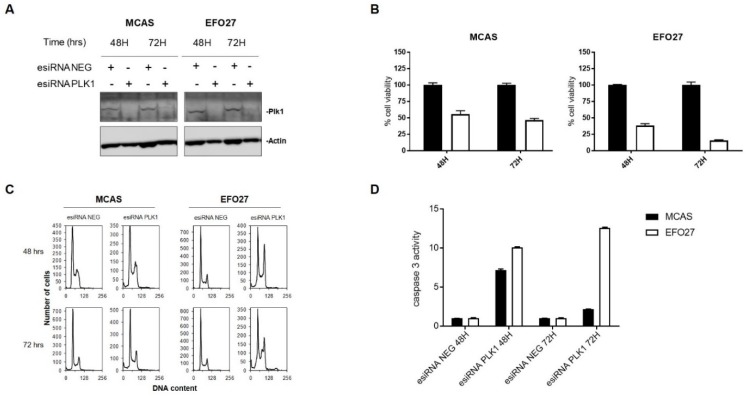
PLK1 downregulation in MCAS and EFO27 cell lines. (**A**) Western blot analysis showing PLK1 downregulation after transfection with esiRNA in MCAS and EFO27 cell lines. (**B**) Percentages of cell viability at 48 and 72 h after esiRNA transfection. Black bar, negative esiRNA; white bar, PLK1 esiRNA. (**C**) Flow cytometric analysis of DNA content. (**D**) Caspase-3 activity in MCAS (black bar) and EFO27 (white bar) cell lines.

To further investigate the effect of downregulation of PLK1, two mucinous ovarian cancer cell lines (MCAS and EFO27) were transiently transfected with negative esiRNA or PLK1 esiRNA, and cell viability, cell cycle phase distribution and apoptosis were evaluated. PLK1 specific esiRNA strongly inhibited the expression of PLK1 in both MCAS and EFO27 (Figure 2A) and caused a strong inhibition in cell growth compared to the cells transfected with negative esiRNA (Figure 2B). Cell cycle analysis showed an accumulation of cells in the G2/M phase and a slight increase in the sub-G1 population 48 and 72 h after esiRNA PLK1 transfection, while no modification of cell cycle distribution was observed after transfection with negative esiRNA (Figure 2C). A small number of polyploid cells, indicative of endoreduplication, were detected at 72 h in EFO27, but not among MCAS cells. Sub-G1 population is suggestive of cell death, and indeed, PLK1 depletion was able to induce apoptosis as measured by activation of caspase-3 (Figure 2D). 

We then moved from esiRNA mediated PLK1 downregulation to its chemical inhibition. To do this, we evaluated the cytotoxic activity of commercially available PLK1 inhibitors (volasertib and onvansertib) as single agents in three mEOC cell lines (MCAS, EFO27, JHOM1) and found both drugs very active at nanomolar concentrations; similar cytotoxic effects were observed in non-mucinous ovarian cancer cells (Table 1). 

Onvansertib treatment in MCAS cells at the dose of IC_50_ induced effects on the cell cycle similar to those observed with esiRNA anti-PLK1. A block in G2/M phase and cell death, suggested by the presence of sub-G1 events, were already present at 24 h and persisted until 48 h. In EFO27 cells, onvansertib treatment led the cells to duplicate their DNA, inhibiting the cytokinesis and inducing the formation of polyploid cells (Figure S1). JHOM1 cells showed a drug induced cell cycle perturbation similar to that induced in MCAS cells. 

### 3.3. Investigation of Synergistic Combination with PLK1 Inhibitors 

**Figure 3:**
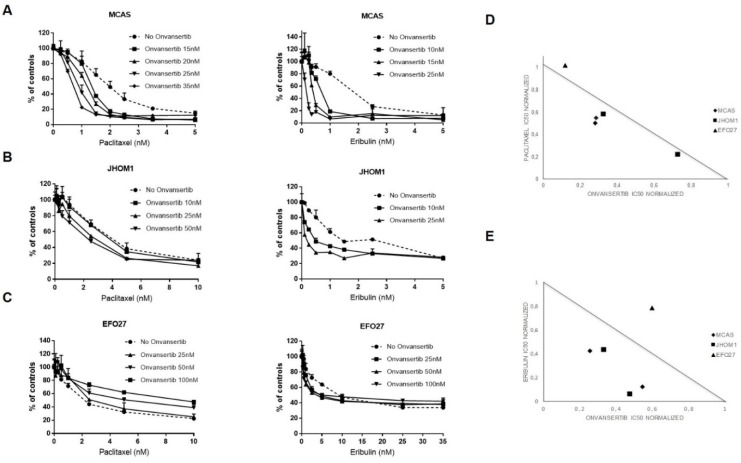
Drug combination of onvansertib and paclitaxel or eribulin in mEOC cell lines. (**A**–**C**) MCAS, JHOM1 and EFO27 treated with non-toxic concentrations of onvansertib and increasing concentrations of paclitaxel and eribulin. (**D**,**E**) normalized IC50 isobolograms showing the synergistic effects of the combination of onvansertib with paclitaxel and eribulin in MCAS and JHOM1 cell lines and the antagonistic effect in EFO27. Each symbol represents an independent experiment.

We then looked for potential synergistic combinations of PLK1 inhibitors with drugs used in first line treatment of ovarian carcinoma (cisplatin and paclitaxel) and with other investigational drugs (eribulin and PI3K inhibitors). No synergism could be found in any of the cell lines, when both PLK1 inhibitors were combined with cisplatin (Figure S2). On the contrary, strong synergism could be observed in two out of the three mEOC cell lines (MCAS and JHOM1 cells) when onvansertib (Figure 3A,B respectively) and volasertib (Figure S3A,B) were combined with both paclitaxel and eribulin, with CI lower than 1 (Figure 3D,E and Figure S3D,E). In EFO27 cell line, the least sensitive of PLK1 inhibitors, no synergistic activity was observed with onvansertib and volasertib (Figure 3C–E and Figure S3C–E). 

MAPK pathway deregulation has been reported in mucinous ovarian cancer, and our cell lines are representative of mEOC in terms of pathway deregulation (Table S3). The combination of volasertib/onvansertib and PIK75 (a PI3K inhibitor) was found to be synergic in all the considered cell lines (Figure S4).

### 3.4. Molecular Studies on the Basis of the Synergistic Onvansertib/Paclitaxel Combination 

**Figure 4:**
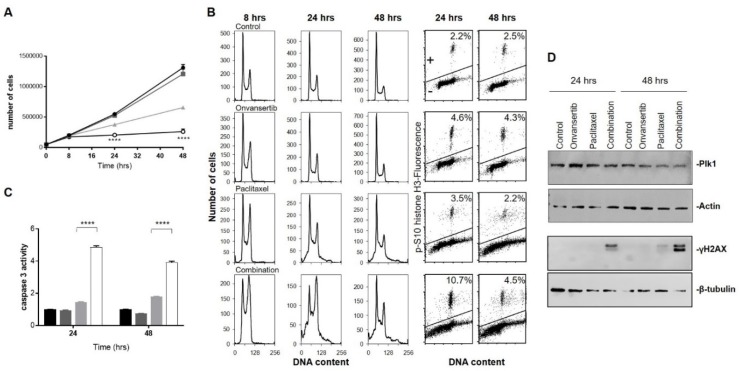
Effect of the combined treatment of onvansertib/paclitaxel on the MCAS cell line. (**A**) Cell growth curves of cells untreated (-●-), treated with 15nM of onvansertib (-■-), 2nM of paclitaxel (-▲-) or the combination (-**○**-). Cell growth was followed from the day of treatment and up to 48 h after treatment. The data are expressed as number of cells ±SD of three replicates. A two-way ANOVA test performed with GraphPad Prism was used for statistical analysis. Statistically significance differences are observed from 24 to 48 h in MCAS cell combined treatment groups vs. control and vs. single agents: **** *p* < 0.0001. (**B**) Analysis of DNA content after 8, 24 and 48 h of treatment with the two drugs either single (paclitaxel 2nM, onvansertib 15nM) or combined, and biparametric analysis of pS10-histone H3 and DNA after 24 and 48 h. Cells detected above the horizontal line were considered as pS10-histone H3-positive, and this percentage was reported in each plot. (**C**) Activation of caspase-3 by enzymatic assay 24 and 48 h after treatment. Data are percentages of untreated cells and represent the means ± SDs. For statistical analysis, a two-way ANOVA test with Bonferroni multiple comparison was used, paclitaxel vs. combination 24 h **** *p* < 0.0001, paclitaxel vs. combination 48 h **** *p* < 0.0001. (**D**) Western blot analysis showing PLK1, γH2AX, actin and beta-tubulin protein levels in MCAS cells protein extracts.

Considering that paclitaxel is a drug approved for the treatment of mucinous ovarian carcinoma, we focused on onvansertib/paclitaxel combination. To investigate the mechanism of the synergistic cytotoxic effect, MCAS cells were treated for 8, 24 and 48 h with low concentrations of onvansertib (15 nM) and paclitaxel (2 nM) alone or in combination. As shown in Figure 4A, the combination caused a marked decrease in cell viability at 24 and 48 h. Onvansertib alone at this concentration induced only a slight accumulation of cells in the G2/M phase after a short treatment time (8 h), while the same effect was more persistent in paclitaxel treated samples. The combined treatment caused a much higher block of cells in the G2/M phase, starting from 8 h and still present at 48 h (Figure 4B). Phospho-histone H3 staining confirmed a higher percentage of cells blocked in M phase of the cell cycle in the combined group compared to single onvansertib and paclitaxel treatment.

The combination treatment caused an increase of sub-G1 population at 24 and 48 h, suggestive of cell death (Figure 4B), which was confirmed by the five-fold increase in caspase-3 activity observed in extracts of cells treated with the two drugs at 24 and 48 h compared to cells untreated or treated with the single agents (Figure 4C). The induction of apoptosis was also investigated at earlier time points, but almost no apoptosis could be seen (Figure S5A). Higher γH2AX levels were observed in cells treated with the combination than in control and single-treatment cells, only at 24 and 48 h (Figure 4D) and not at the earlier time points investigated (Figure S5B), suggesting that its increase is related to apoptosis induction. Similar results were obtained with the combination of onvansertib and eribulin at 48 h (Figure S6). We looked for possible pharmacodynamic markers of activity. As shown in Figure 4D, no difference was observed in PLK1 protein levels after treatment, either after single or combined treatments. 

### 3.5. In Vivo Effects of the Combination

**Figure 5:**
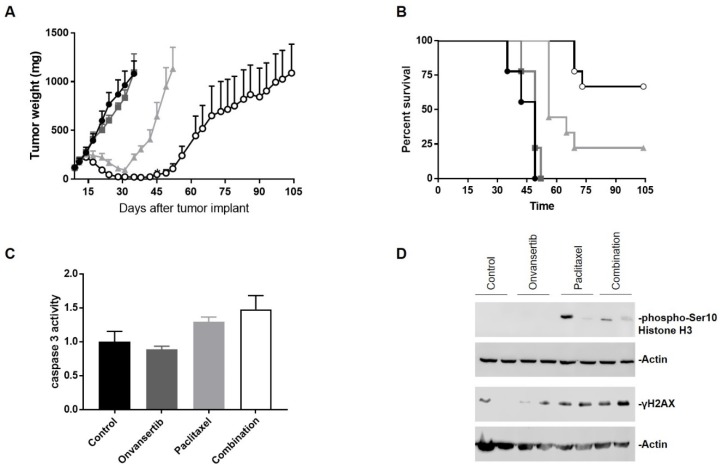
Antitumor effect and target modulation of the combined treatment in MCAS xenografts. (**A**) Tumor growth curves of MCAS xenograft control (-●-) and treatments with onvansertib (-■-), paclitaxel (-▲-) or both (-**○**-). Data are represented as means ± SEs. For statistical analysis, two-way ANOVA test with Bonferroni multiple comparison was used. On day 35 the significant differences were as follows: control vs. paclitaxel and combination, ****, *p* < 0.0001; onvansertib vs. paclitaxel and combination, ****, *p* < 0.0001. One-way ANOVA test for repeated measures with Bonferroni multiple comparison was used for statistical analysis at day 45 and the significant differences were as follows: control vs. combination, **, *p* < 0.01; onvansertib vs. combination, ****, *p* < 0.0001; paclitaxel vs. combination, *, *p* < 0.05. (**B**) Kaplan–Meier survival curves of mice. Log-rank test (Mantel–Cox) was used to calculate p-values comparing the survival curves. Control vs. paclitaxel and combination ****, *p* < 0.0001; onvansertib vs. paclitaxel and combination ****, *p* < 0.0001; paclitaxel vs. combination **, *p* < 0.01. (**C**) Caspase 3 activity in tumor tissue extracts from mice treated as described in the methods. Data are percentages of untreated cells and represent the means ± SDs of two independent experiments each. One-way ANOVA test with Tukey’s multiple comparison was used, and the differences were as follows: control vs. combination *, *p* < 0.05; onvansertib vs. paclitaxel *, *p* < 0.05; onvansertib vs. combination **, *p* < 0.01. (**D**) Western blot analysis showing pS10 Histone H3 and γH2AX protein levels in xenograft tumors protein extracts. Two replicates for each condition were used.

On the basis of the strong in vitro synergistic effect, the combination onvansertib and paclitaxel was tested in vivo in nude mice transplanted with MCAS cells. To try to magnify the effect of the combination, we used a relatively low dose of onvansertib (50 mg/kg); indeed, at this dose, as a single agent the drug was inactive (T/C = 78.5%) and no tumor growth inhibition was observed (Figure 5A); paclitaxel at the dose of 20 mg/kg was able to induce tumor growth inhibition and some regressions. However, the combination of the two drugs, which was well tolerated (Figure S7), was much more active than paclitaxel as a single agent, as suggested by a stronger and more sustained tumor regression and tumor growth inhibition (Figure 5A), which translated in a longer survival time (Figure 5B). Pharmacodynamic analysis on tumors treated with drugs as single agents or a combination at the same doses used in the activity, showed a slight increase of caspase-3 activation in tumor protein extracts from mice treated with the combination (Figure 5C) and a slight increase of the mitotic marker phospho-histone H3 and γH2AX after both paclitaxel and combined treatments (Figure 5D).

## 4. Discussion

Advanced mucinous ovarian cancer is a chemo-resistant disease with a poor clinical outcome [6,7,23]. Therefore, the development of more specific and active therapeutic strategies for late stage mEOC are required to improve the survival of these patients. In order to find new therapeutic options for this rare cancer, we performed a high-throughput screening using a human siRNA kinase library in a mEOC cell line to identify genes interfering with cell growth/viability. After two independent screenings and validation experiments using specific esiRNAs, PLK1, belonging to the family of mitotic serine/threonine kinases, was identified as the most significant kinase whose downregulation caused a strong growth inhibiting effect in MCAS cell line.

PLK1 is the most investigated member of human PLK family and is essential for mitosis, as it regulates several stages of this phase of cell cycle (mitotic entry, centrosome maturation, mitotic spindle formation, transition from metaphase to anaphase and cytokinesis) [24,25]. Several studies have shown that PLK1 is overexpressed in a broad spectrum of malignant tumors, including ovarian cancer, and its expression correlates with histological grade and poor prognosis [26,27]. For all these reasons, in recent years, PLK1 has become an interesting therapeutic target in oncology [28,29,30], and several PLK1 inhibitors have indeed been developed and are under clinical investigation [31], including volasertib in phase III study in acute myeloid leukemia [32], and onvansertib, undergoing investigation in phase II in combination with other drugs (https://trovageneoncology.com/pipeline/). Of note, a recent study reported hints of antitumor activity in patients with platinum-resistant or -refractory ovarian cancer treated with volasertib, suggesting PLK1 could be an interesting target to be further investigated in ovarian cancer [33]. 

Based on the screening results, the specific inhibition of PLK1 with esiRNA in mEOC cell lines leads to cell growth inhibition, G2/M cell cycle blocking, induction of apoptosis and endoreduplication. While these data are consistent with previous findings of the inhibition of PLK1 using RNAi [34,35,36], this is the first observation that PLK1 inhibition is effective in mEOC. In addition, PLK1 inhibitors volasertib and onvansertib were cytotoxic at low nanomolar concentrations in all the mEOC cell lines tested, similarly to what has been reported in other tumor cells [37,38,39]. When we investigated the effect of volasertib and onvansertib treatment in combination with the chemotherapeutic drugs used as standard therapy for treatment of mEOC, we did not find synergy with cisplatin in any of the mEOC cell lines tested; on the contrary, a synergistic effect was observed when onvansertib/volasertib were combined with microtubule-interacting drugs, paclitaxel and eribulin, in two out of three cell lines tested. The strong effect of the drug combination is demonstrated by a CI<1, which corroborates a synergistic drug interaction. The lack of synergy in EFO27 cell line could be due to its lower sensitivity to both volasertib and onvansertib, as indicated by the 3–6 fold higher drug IC50s compared to the other mEOC cell lines. Some evidence suggests that high concentrations of PLK1 inhibitors might promote mitotic slippage favoring cell survival [40]. In time lapse experiments, EFO27 cells treated with onvansertib at different concentrations entered mitosis, but failed to undergo chromosome segregation and cytokinesis with the formation of polyploid and multinucleated cells (endoreduplicated cells) that might be more resistant to apoptosis induced by chemotherapy (Figure S1A, time lapse experiments—data not shown). In support of these data, when we tested the induction of apoptosis after paclitaxel/onvansertib treatment in EFO27 cell line, a much lower increase of apoptosis was observed when compared to MCAS cell lines (Figure S8). The lack of synergism with cisplatin is not surprising. The two drugs act on different targets in different phases of the cell cycle, and the G2 block induced by cisplatin would possibly downregulate the number of cells in M phase where onvansertib should maximize its activity. 

We tried to elucidate the underlying molecular mechanisms of the synergism of PLK1 inhibitors with paclitaxel and found that the combined treatment causes strong growth inhibition and mitotic arrest, which triggers activation of caspase-3 and apoptosis; these results correlate with previous findings [36,41]. Recently, Noack *et al*. identified a synthetic lethality between volasertib and paclitaxel in ovarian cancer cell lines with CCNE1-amplification, demonstrating that the combination induces a prolonged mitotic arrest and cell death, shifting the balance between pro- and anti-apoptotic proteins toward cell death [42]. 

Our in vitro data translated into a pronounced antitumor activity of the combination in the MCAS mEOC xenograft with a lack of evident toxicity. The reported in vitro molecular mechanisms were also observed in vivo, where in tumors treated with the combination showed a higher level of phospho-histone H3, indicative of a sustained mitotic block and a higher induction of γH2AX, indicative of increased DNA damage, likely due to apoptosis induction. To exclude direct DNA damage causing an increased in γH2AX, we looked for the induction of ROS in our experimental conditions (single and combined treatments). However, we did not see any ROS production (data not shown), suggesting that apoptosis is likely to be responsible for the observed γH2AX induction. 

The relative lack of toxicity is due to the relatively low dosage of both drugs used, which again underscores the synergistic activity of the combination. In addition, even with the use of higher doses in a clinical setting, the spectrum of the reported side effects of the single drugs (hamatological toxicity for onvansertib and neurotoxicity for paclitaxel) [43,44] are not overlapping, fostering this drug combination. We also reported a synergistic activity when PLK1 inhibitors were combined with both eribulin and PI3K inhibitor. The synergistic effect with eribulin, an anti-mitotic drug with a mechanism of action different from paclitaxel [45], has already been reported in rhabdomyosarcoma [46] and Ewing sarcoma [47]. The latter data reinforce the potentiality of the combination between PLK1 inhibitors and the class of anti-mitotic drugs. The synergistic activity of PLK1 and PI3K inhibition combination has been recently reported in anaplastic thyroid cancer models and was likely to be due to a reducing mitotic slippage and endoreduplication [48]. Finally, considering that PLK1 inhibitor shows activity also in HGSOC, the combination of PLK1 inhibitor and paclitaxel could represent a new option also in this setting, particularly in platinum refracting or relapsing tumors. 

## 5. Conclusions

Taken together, our in vitro and in vivo data indicate, for the first time, that PLK1 inhibitors in combination with antimitotic drugs might represent potential therapeutic options for the treatment of mEOC patients. Considering that paclitaxel is already approved for mEOC treatment, this could favor a speedy clinical translation of this combination. 

## Figures and Tables

**Table 1:** Onvansertib and volasertib IC50s (nM) in the different ovarian cancer cell lines.

Mucinous Cell Lines	Onvansertib (nM)	Volasertib (nM)
MCAS	48 ± 6	16 ± 2
EFO27	287.9 ± 22	71 ± 7
JHOM1	87.7 ± 11	13.6 ± 4
Serous cell lines		
A2780	47 ± 4.8	13.5 ± 1.9
OVCAR5	65.9 ± 16	28.8 ± 5.6
SKOV3	132.1 ± 25	44.2 ± 11

## Supplementary Materials

The following are available online at https://www.mdpi.com/2072-6694/12/3/672/s1, Figure S1: cell cycle analysis of mEOC cells. Figure S2: drug combination of onvansertib or volasertib and cisplatin in mEOC cell lines. Figure S3: drug combination of volasertib and paclitaxel or eribulin in mEOC cell lines. Figure S4: drug combination of onvansertib or volasertib and PIK75 in mEOC cell lines. Figure S5: DNA damage and apoptosis kinetics after combination of onvansertib/paclitaxel. Figure S6: effect of the combined treatment onvansertib/eribulin on MCAS cell line. Figure S7: body weight curves in MCAS xenografts. Figure S8: caspase-3 activity assay in EFO27 cell line after combination of onvansertib/paclitaxel. Table S1: Drugs and mechanism of action. Table S2: hits identified with a mean of T/C ≤ 0.6 in the two experiments. Table S3: Mutation status of mEOC cell lines.

## Abbreviations

EOC:	epithelial ovarian cancer
mEOC:	mucinous epithelial ovarian cancer
HGSOC:	high grade serous ovarian cancer
RNAi:	RNA interference
PLK1:	polo-like kinase 1
esiRNA:	endoribonuclease-prepared siRNAs
γH2AX:	phospho histone family member 2AX
