# Supplementary material for: Identification of PLK1 as a New Therapeutic Target in Mucinous Ovarian Carcinoma
Source: Cancers (Basel). 2020 Mar 13;12(3):672. doi: 10.3390/cancers12030672 (PMC7140026; doi:10.3390/cancers12030672)
Supplement: Supplementary file 1 [file cancers-12-00672-s001.zip › Supplementary Figures.pptx]

## Slide 1
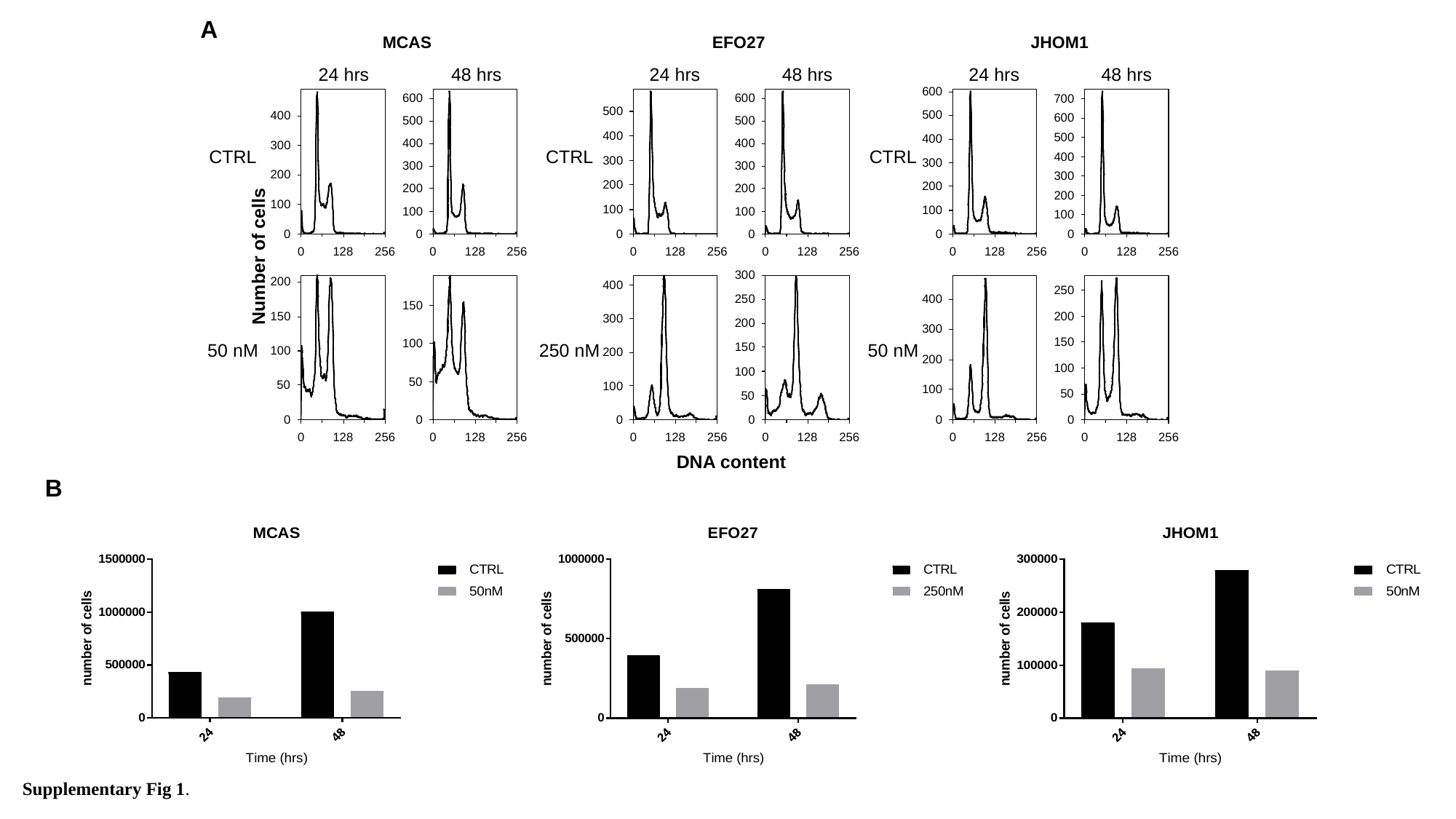

A
MCAS
EFO27
JHOM1
24 hrs
48 hrs
24 hrs
48 hrs
24 hrs
48 hrs
CTRL
CTRL
CTRL
Number of cells
50 nM
250 nM
50 nM
DNA content
DNA content
DNA content
DNA content
B
Supplementary Fig 1.

## Slide 2
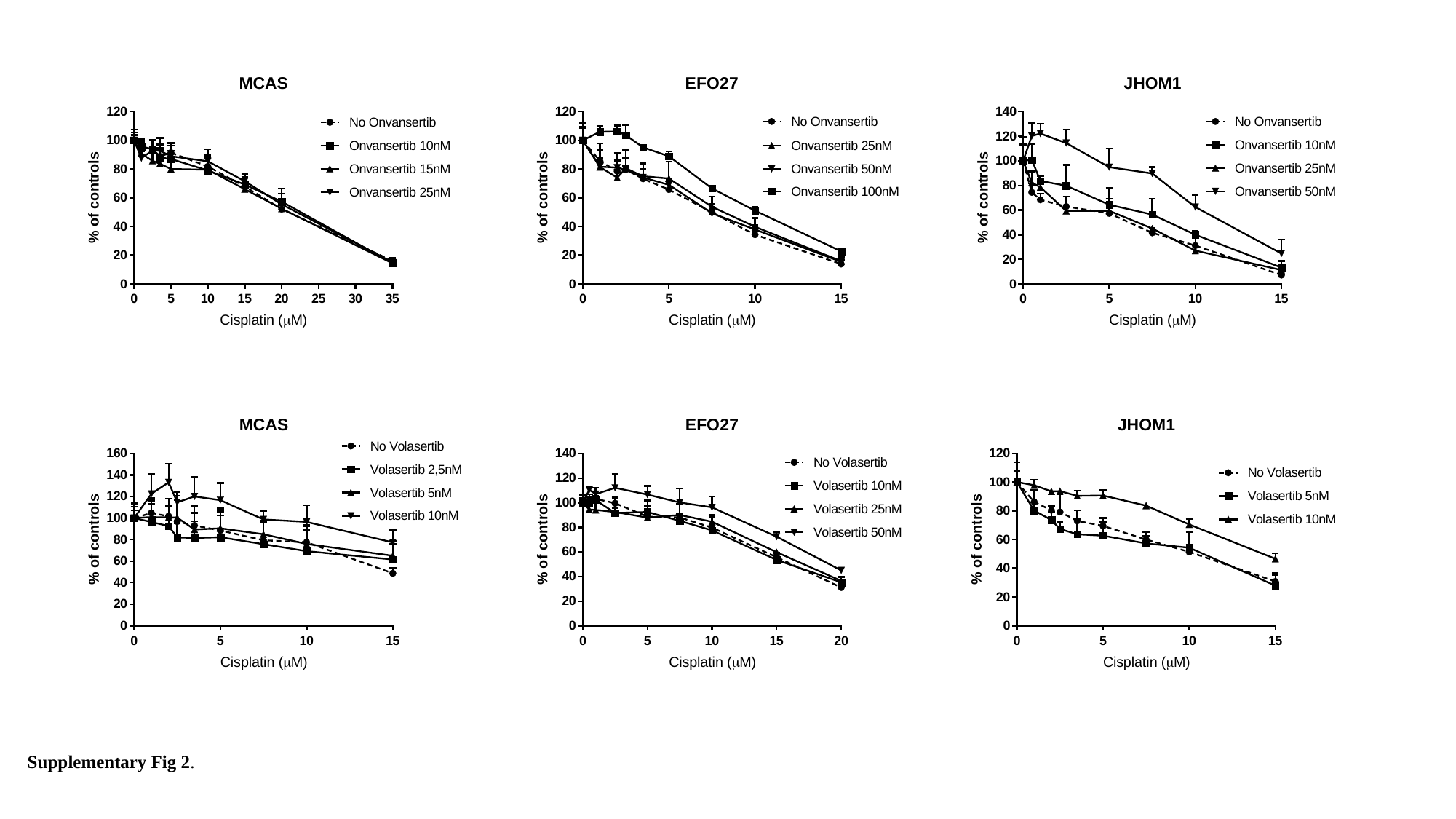

Supplementary Fig 2.

## Slide 3
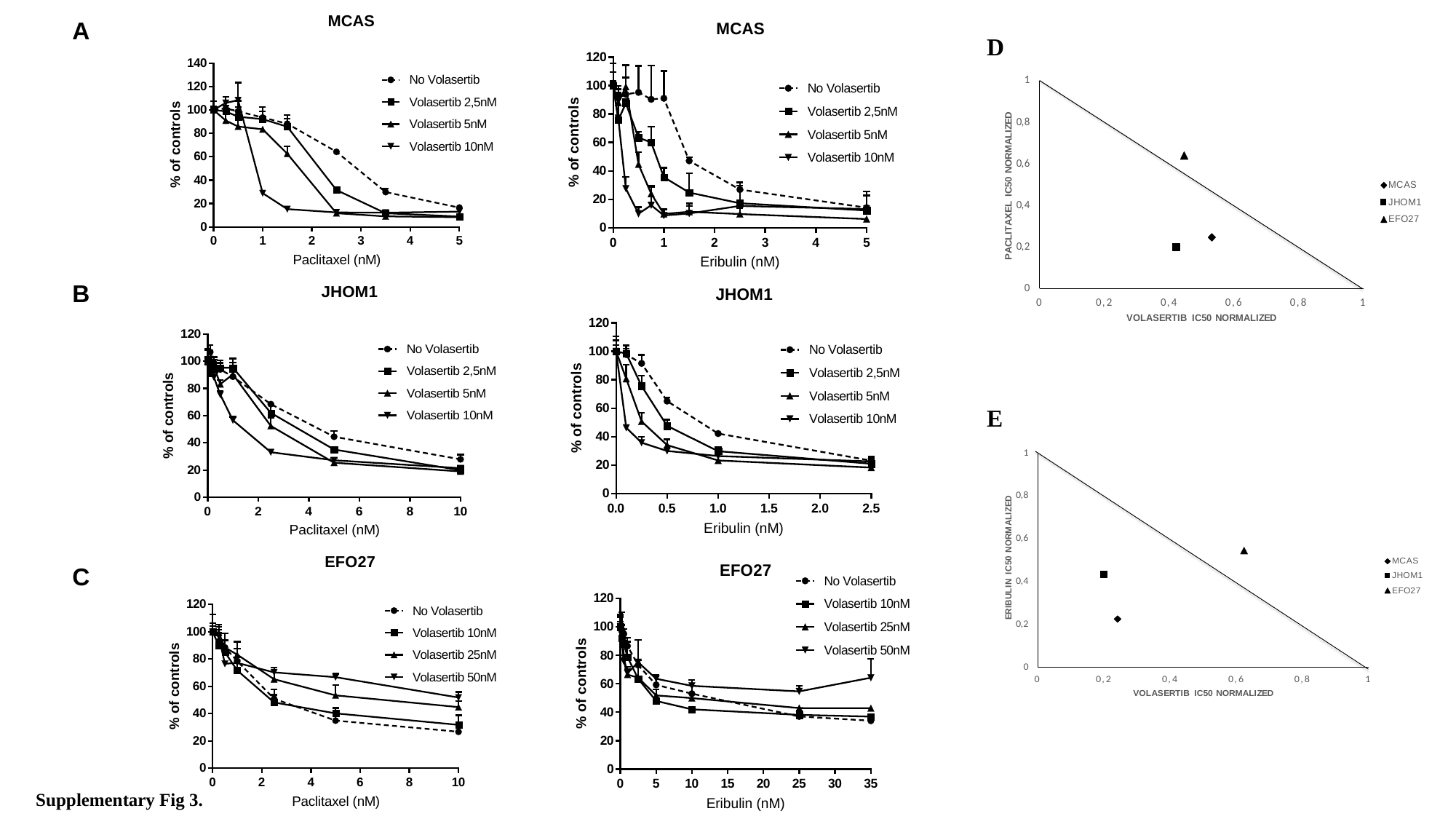

A
D
B
E
C
Supplementary Fig 3.

## Slide 4
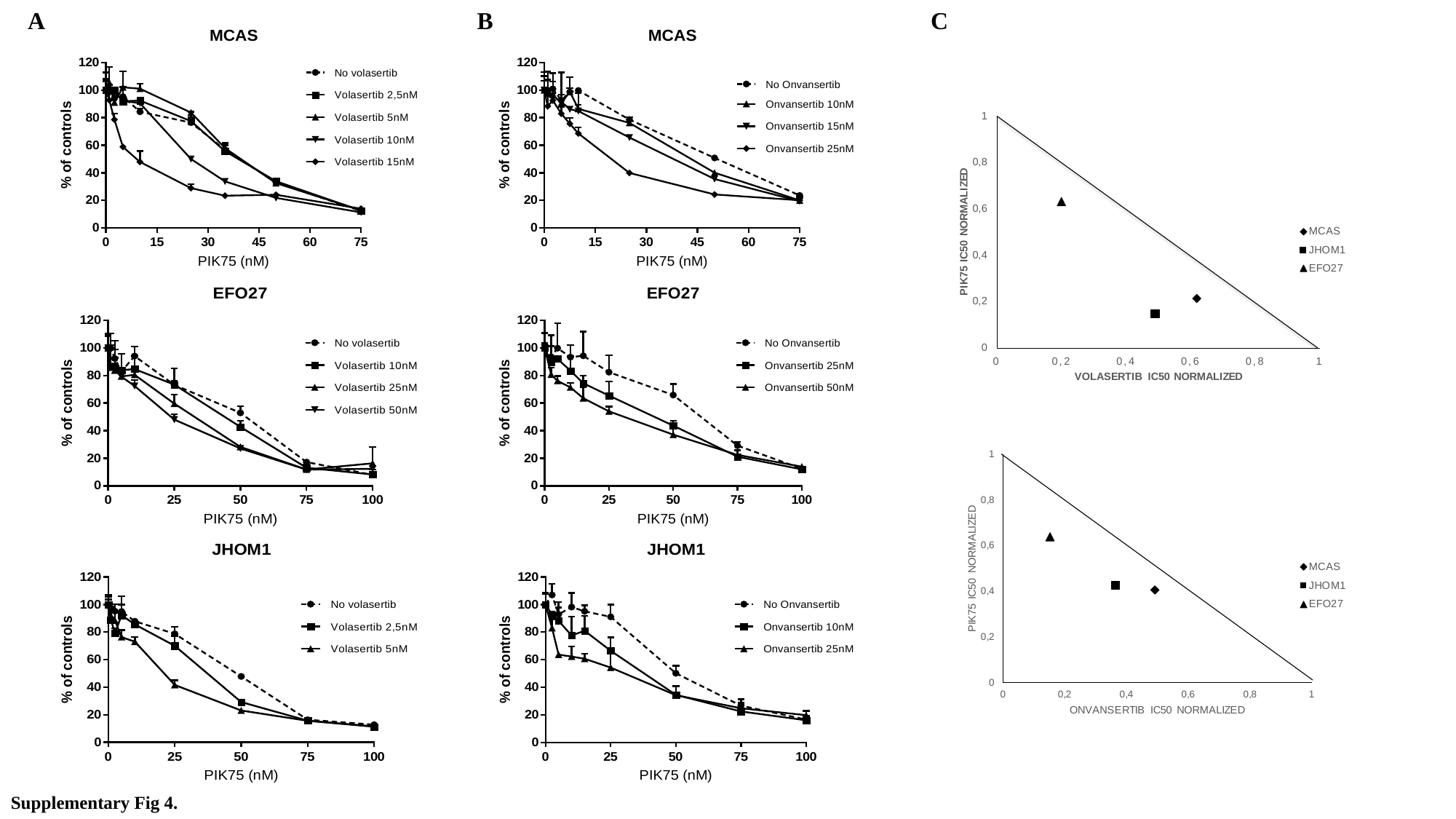

A
B
C
Supplementary Fig 4.

## Slide 5
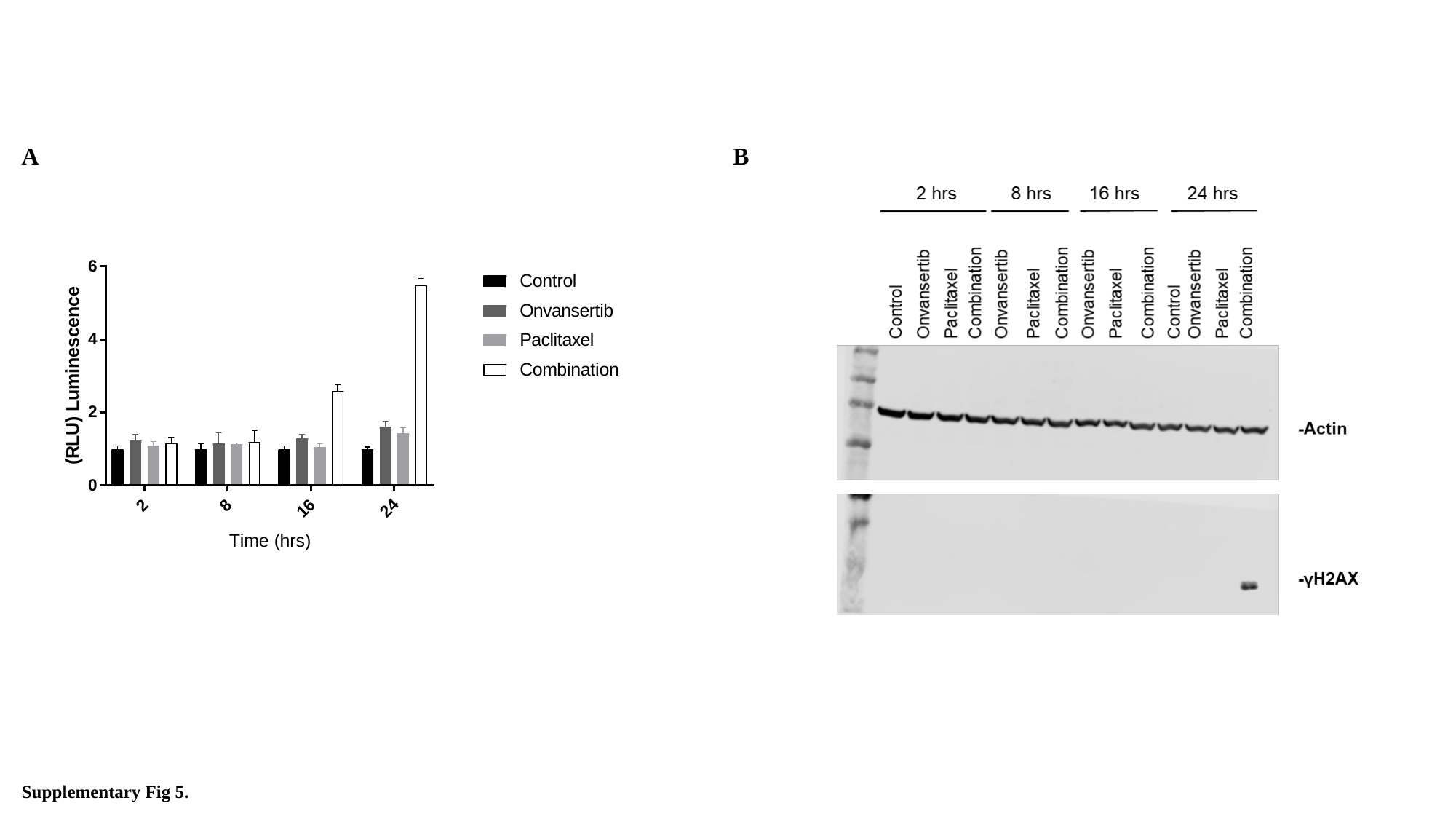

A
B
Supplementary Fig 5.

## Slide 6
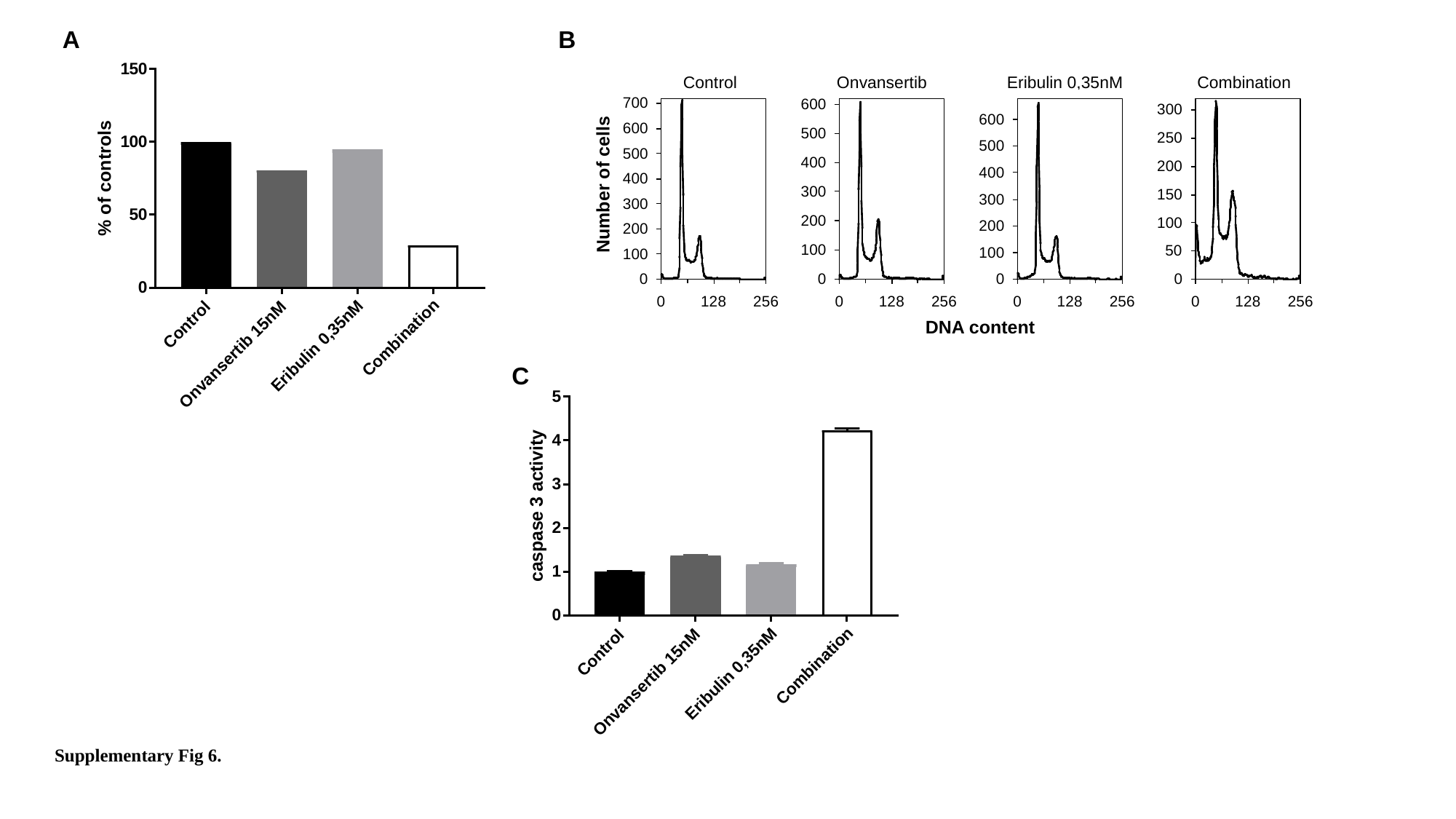

A
B
Control
Onvansertib 15nM
Eribulin 0,35nM
Combination
Number of cells
DNA content
C
Supplementary Fig 6.

## Slide 7
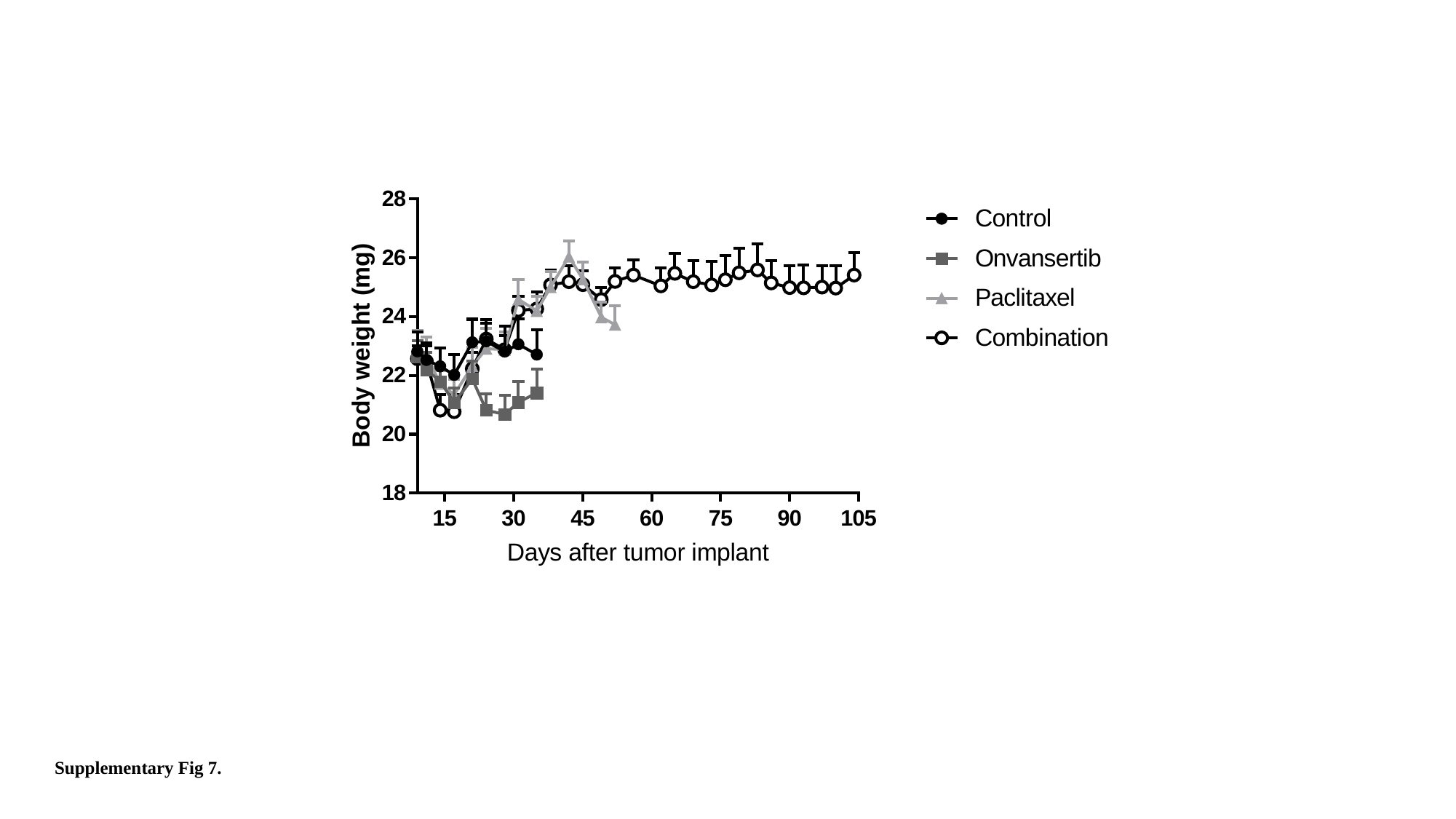

Supplementary Fig 7.

## Slide 8
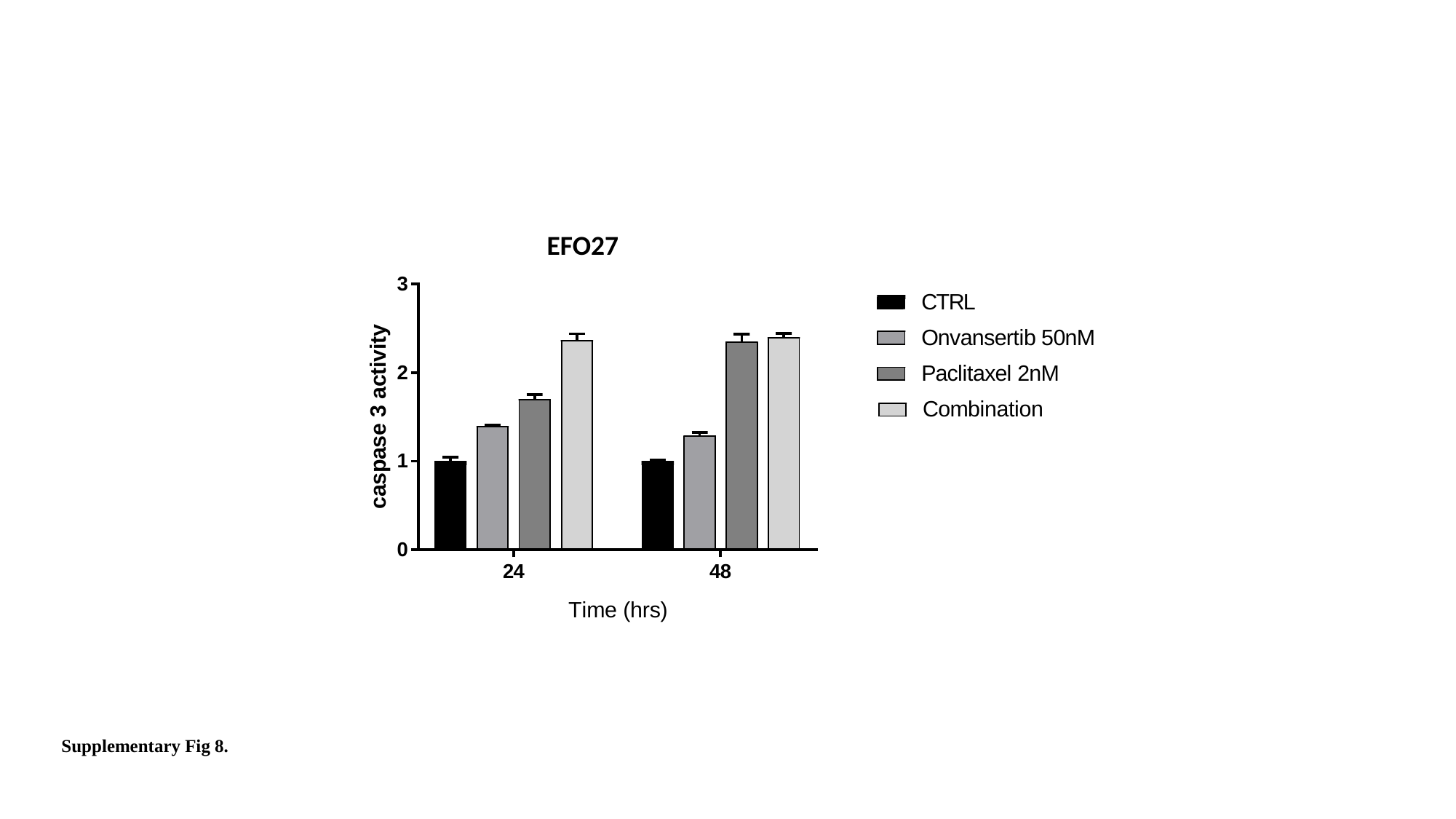

EFO27
Supplementary Fig 8.
