# Supplementary material for: Identification of PLK1 as a New Therapeutic Target in Mucinous Ovarian Carcinoma
Source: Cancers (Basel). 2020 Mar 13;12(3):672. doi: 10.3390/cancers12030672 (PMC7140026; doi:10.3390/cancers12030672)
Supplement: Supplementary file 1 [file cancers-12-00672-s001.zip › Legends to Supplementary Figure.docx]

**LEGENDS TO SUPPLEMENTARY FIGURES.**

**Supplementary Fig 1**. (A) **Cell cycle analysis of mEOC cells**. MCAS, EFO27 and JHOM1 were treated with onvansertib at the IC50 dose. Analysis of DNA content was performed after 24 and 48 hrs of treatment. (B) Cell growth of MCAS, EFO27 and JHOM1 untreated or treated with onvansertib at the IC50 dose.

**Supplementary Fig 2**. **Drug combination of onvansertib or volasertib and cisplatin in mEOC cell lines**. MCAS, EFO27 and JHOM1 treated with non toxic concentration of volasertib or onvansertib and growing concentration of cisplatin.

**Supplementary Fig 3. Drug combination of volasertib and paclitaxel or eribulin in mEOC cell lines**. (A, B, C) MCAS, JHOM1 and EFO27 treated with non toxic concentration of volasertib and growing concentration of paclitaxel and eribulin. (D, E) normalized IC50 isobologram showing the synergistic effects of the combination of volasertib with paclitaxel and eribulin in MCAS and JHOM1 cell lines and the antagonistic effect in EFO27.

**Supplementary Fig 4. Drug combination of onvansertib or volasertib and PIK75 in mEOC cell lines**. (A) MCAS, EFO27 and JHOM1 treated with non toxic concentration of volasertib and growing concentration of PIK75. (B) MCAS, EFO27 and JHOM1 treated with non toxic concentration of onvansertib and growing concentration of PIK75. (C) Normalized IC50 isobologram showing the synergistic effects of the combination in mEOC cell lines.

**Supplementary Fig 5.** (A) Caspase 3/7 activity in MCAS cells untreated or treated with 15nM of onvansertib, 2nM of paclitaxel or the combination for 2, 8, 16 and 24 hrs, detected by the Caspase-Glo 3/7 assay (Promega). Data are relative light units (RLU) normalized to control and represent the mean of 5 replicates ± standard deviation. (B) Western blot analysis showing γH2AX and actin protein levels in MCAS protein extracts of cells treated with the same schedule as described in A.

**Supplementary Fig 6**. (A) Cell growth of MCAS untreated, treated with 15nM of onvansertib, 0,35nM of eribulin or the combination for 48 hrs. (B) Flow cytometric analysis of DNA content after 48hrs of treatment with the two drugs either singly or combined. (C) Caspase-3 activity after 48hrs of treatment. Data are percentage of untreated cells and represent the mean ± SD.

**Supplementary Fig 7.** Body weight curves in MCAS xenografts treated with onvansertib, paclitaxel or both. Data are represented as mean ± SE.

**Supplementary Fig 8**. Activation of caspase-3 by enzymatic assay 24 and 48 hrs after treatment in EFO27 cell line. Data are percentage of untreated cells and represent the mean ± SD.
