# Supplementary material for: Identification of PLK1 as a New Therapeutic Target in Mucinous Ovarian Carcinoma
Source: Cancers (Basel). 2020 Mar 13;12(3):672. doi: 10.3390/cancers12030672 (PMC7140026; doi:10.3390/cancers12030672)
Supplement: Supplementary file 1 [file cancers-12-00672-s001.zip › Table S1.docx]

| **Drugs** | **Target** |
| --- | --- |
| Onvansertib | PLK1 inhibitor |
| Volasertib | PLK1 inhibitor |
| Cisplatin | DNA damaging agent |
| Paclitaxel | microtubule-stabilizing drug |
| Eribulin | Microtubule-targeting agents |
| PIK75 | PI3K p110α inhibitor |

**Table S1. Drugs and mechanism of action.**
