# Supplementary material for: Identification of PLK1 as a New Therapeutic Target in Mucinous Ovarian Carcinoma
Source: Cancers (Basel). 2020 Mar 13;12(3):672. doi: 10.3390/cancers12030672 (PMC7140026; doi:10.3390/cancers12030672)
Supplement: Supplementary file 1 [file cancers-12-00672-s001.zip › Table S3.docx]

| **GENE** | **MCAS** | **EFO27** | **JHOM1** |
| --- | --- | --- | --- |
| *TP53* | T22S | R273C | R213 |
| *KRAS* | G12D | WT | WT |
| *PIK3CA* | H1047R | H510N | WT |
| *PTEN* | WT | L265fs | L318fs |
| *ERBB2* | WT | V842I | WT |
| *ARID1A* | WT | R1722 | WT |
| *BRAF* | WT | WT | WT |

**Table S3. Mutation status of mEOC cell lines.**

Data were retrieved from Broad Institute Cancer Cell Line Encyclopedia (CCLE).
