# Supplementary figures and images for: Identification of PLK1 as a New Therapeutic Target in Mucinous Ovarian Carcinoma
Source: Cancers (Basel). 2020 Mar 13;12(3):672. doi: 10.3390/cancers12030672 (PMC7140026; doi:10.3390/cancers12030672)

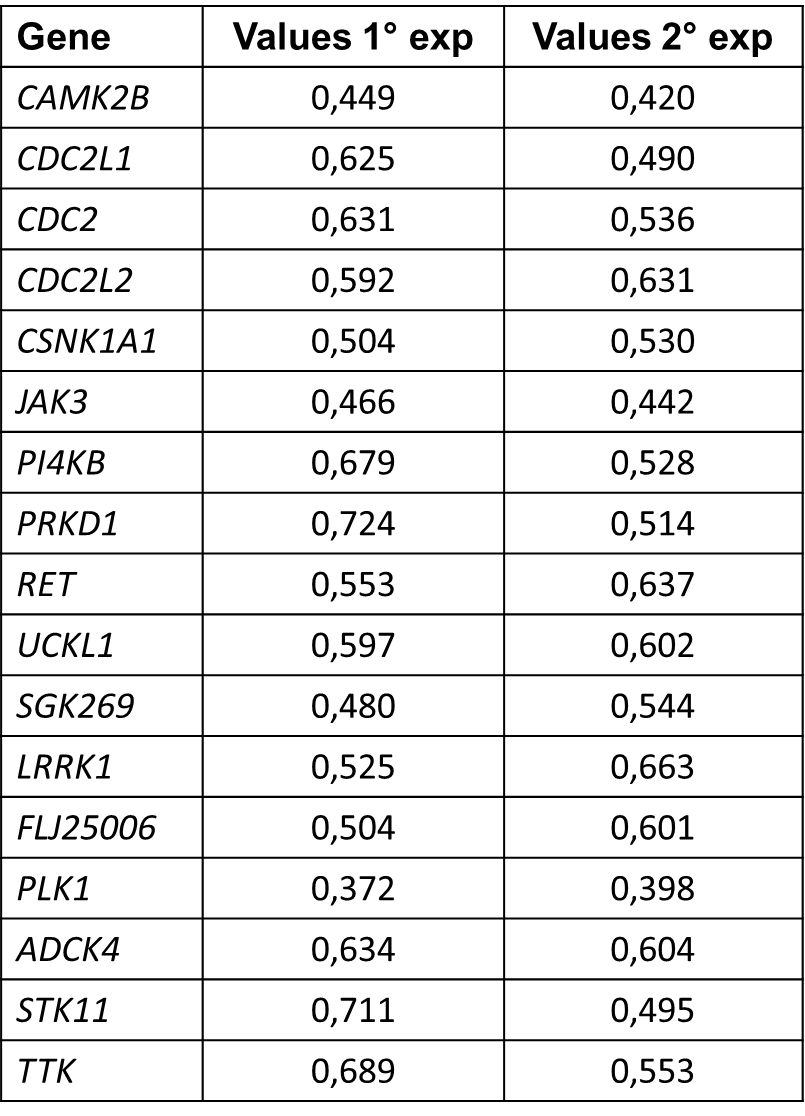


**Table S2. Hits identified with a mean of T/C ≤ 0.6 in the two experiments.**

Supplement: Supplementary file 1 [file cancers-12-00672-s001.zip › Table S2.docx]
